# Phylogenomic analysis of cytochrome P450 multigene family and its differential expression analysis in pepper (*Capsicum annuum* L.)

**DOI:** 10.3389/fpls.2022.1078377

**Published:** 2022-12-06

**Authors:** Yupeng Hao, Zeyu Dong, Yongyan Zhao, Wenchen Tang, Xueqiang Wang, Jun Li, Luyao Wang, Yan Hu, Lei Fang, Xueying Guan, Fenglin Gu, Ziji Liu, Zhiyuan Zhang

**Affiliations:** ^1^ Hainan Institute, Zhejiang University, Sanya, China; ^2^ Spice and Beverage Research Institute, Sanya Research Institute, Chinese Academy of Tropical Agricultural Sciences/Hainan Key Laboratory for Biosafety Monitoring and Molecular Breeding in Off-Season Reproduction Regions, Sanya, China; ^3^ Tropical Crops Genetic Resources Institute, Chinese Academy of Tropical Agricultural Sciences/Key Laboratory of Crop Gene Resources and Germplasm Enhancement in Southern China, Ministry of Agriculture, Haikou, China; ^4^ Zhejiang Provincial Key Laboratory of Crop Genetic Resources, Institute of Crop Science, Plant Precision Breeding Academy, College of Agriculture and Biotechnology, Zhejiang University, Hangzhou, China

**Keywords:** cytochrome P450, gene family, pepper, evolution, phylogenetic analysis

## Abstract

Plant cytochrome P450 is a multifamily enzyme widely involved in biochemical reactions for the synthesis of antioxidants, pigments, structural polymers, and defense-related compounds. Pepper (*Capsicum annuum* L.) is an economically important plant. A comprehensive identification and characterization of P450 genes would provide valuable information on the evolutionary relationships of genes and their functional characteristics. In this study, we identified P450 genes in pepper with the aid of bioinformatics methods to investigate the phylogenetic relation, gene structure, chromosomal localization, duplicated events, and collinearity among Solanaceae species. We identified and classified 478 genes of P450 from the pepper genome into two major clades and nine subfamilies through phylogenetic analysis. Massive duplication events were found in the P450 gene family, which may explain the expansion of the P450 gene family. In addition, we also found that these duplication genes may have undergone strict purification selection during evolution. Gene expression analysis showed that some P450 genes that belong to clan 71 in pepper may play an important role in placenta and pericarp development. Through quantitative real-time polymerase chain reaction and transcriptome analysis, we also found that many P450 genes were related to defensive and phytohormone response in pepper. These findings provide insight for further studies to identify the biological functions of the P450 genes in pepper.

## Introduction

Cytochrome P450 (P450) is one of the largest superfamilies of enzymes and is named for the absorption band at 450 nm of its carbon-monoxide-bound form (Werck-Reichhart and Feyereisen, 2000). The first P450 gene was found in rat liver microsomes (Omura and Sato, 1962; Klingenberg, 2003). The first P450 X-ray crystal structure was obtained from bacterial P450_cam_ (Poulos et al., 1985). Subsequently, as an increasing number of P450 genes were discovered, the subfamily of P450 genes was also expanding (Nelson, 2006; Guttikonda et al., 2010; Bak et al., 2011). The P450 family is present in almost all organisms, including plants, insects, animals, fungi, bacteria, and viruses (Nelson, 2009). More than 300,000 P450 genes have been identified in different organisms, including approximately 16,000 plant P450 genes (Nelson, 2018). The P450 family is the third-largest gene family present in *Arabidopsis* (Morant et al., 2003), and it plays an important role in the synthesis of antioxidants (He et al., 2017), phytohormones (Bishop and Koncz, 2002), structural polymers, and defense-related compounds in plants (Hofer et al., 2008; Franke et al., 2012; Koch et al., 2013; Ma and Tredway, 2013; Ghosh, 2017; Yu et al., 2017). In recent years, an increasing number of genes related to stress resistance have been identified, among which the P450 genes have been widely studied (Wang et al., 2020b; Sun et al., 2021). Some P450 genes could be involved in secondary metabolism, such as the gossypol biosynthesis pathway in cotton (Tian et al., 2018; Huang et al., 2020). *CYP86A2*, which is involved in the biosynthesis of epidermal lipids, has conferred drought tolerance in *Arabidopsis* (Xiao et al., 2004). *CsCYP75B1* was involved in antioxidant flavonoid metabolism and regulated drought tolerance by enhancing reactive oxygen species scavenging activity in citrus (Rao et al., 2020). In perennial ryegrass and tall fescue, P450 genes showed a strong response to cold stress (Tao et al., 2017). Two genes encoding cytochrome P450 proteins were also upregulated in the transcriptome profiling of roses under cold stress treatment (Dos Reis et al., 2020). In *Avicennia officinalis* and *Arabidopsis*, *CYP94B1* has been associated with plant salt tolerance by a helping plant to form an apoplastic barrier (Krishnamurthy et al., 2020). P450 genes were also found to be involved in the biosynthesis of jasmonic acid (JA), including *GmCYP82A3* in soybean and *DzCYP72As* in *Dioscorea zingiberensis* (Yan et al., 2016). In addition, P450 genes also played a crucial role in resistance to biotic stress. Transgenic tobacco overexpressing *GmCYP82A3* showed high resistance to black shank (*Phytophthora parasitica*) and gray mold (*Botrytis cinerea*), and the P450 gene *PAD3* in *Arabidopsis* has been shown to enhance the resistance to green peach aphid (*Myzus persicae*) (Prince et al., 2014).

According to the evolutionary relationship of P450 genes in different organisms, the P450 genes in plants can generally be divided into two categories: A type and non-A type. The A-type subfamily is a plant-specific P450 that evolved from the differentiation of plants, whereas the non-A-type subfamily has a sequence similarity with animals and fungi (Yu et al., 2017). In *Arabidopsis*, the A type includes the CYP71 subfamily, and the non-A type has eight different subfamilies, namely, CYP51, CYP72, CYP710, CYP711, CYP74, CYP85, CYP86, and CYP97 (Bak et al., 2011). Gene duplication is considered to be one of the primary driving forces in the evolution of genomes and genetic systems (Moore and Purugganan, 2003). Tandem and segmental gene duplication can produce a large number of gene families (Qiao et al., 2019). Tandem duplication was characterized as having multiple members of one family occurring within the same intergenic region or in neighboring intergenic regions (Blanc and Wolfe, 2004; Li et al., 2012). Segmental duplication of multiple genes through polyploidy may be followed by chromosome rearrangements (Vollger et al., 2022). These research methods provide key information for us to study the phylogeny of the cytochrome P450 gene family.

Pepper (*Capsicum annuum* L.) is an essential crop in the Solanaceae family. Its fruits are the second most consumed vegetable worldwide (Airaki et al., 2012). Capsaicinoids are secondary metabolites of phenolic compounds that impart an appealing pungent taste to pepper fruits and have a variety of proven health benefits and industrial applications (Naves et al., 2019). In addition, pepper is sensitive to various abiotic and biotic stresses—in particular, extreme environments have seriously affected the growth and development of pepper, resulting in a decline in the yield and quality of pepper (Feng et al., 2019; Ramzan et al., 2021). The P450 gene family is widely involved in plant development and response to various stresses. A comprehensive analysis of the P450 gene family in pepper, however, has not been conducted. Next-generation sequencing methods provide an ideal tool for elucidating the P450 genes in pepper. In this study, we used the bioinformatics methods to analyze the number, chromosomal localization, replication events, phylogeny, and gene expression as well as provided a panoramic view of the P450 gene family in pepper. These results established a solid foundation for further genetic studies on the biological function of P450 in pepper, particularly in relation to stress tolerance.

## Materials and methods

### Plant material

Two weeks after germination, we maintained the pepper (*C. annuum* cv. CM334) seedlings in a growth room at 24°C ± 1°C with a 16-h light and an 8-h dark photoperiod. At the six-true-leaf stage, we subjected young plants to a temperature of 10°C or 40°C to mimic cold or heat stress. The third or fourth leaves were harvested per replicate at 0, 3, 6, 12, 24, and 72 h after treatment. We collected three biological replicates at each time point per condition. The leaves were flash-frozen in liquid nitrogen and stored in an ultra-low-temperature refrigerator at −80°C for RNA extraction and quantitative real-time polymerase chain reaction (qRT-PCR).

### Identification of the CaCYP genes

We obtained the genome data of *C. annuum* cv. CM334 from the pepper website ^1^ and downloaded other genome files, such as *Arabidopsis thaliana* TAIR10, *Solanum lycopersicum* ITAG4.0, and *Solanum tuberosum* v6.1, from Phytozome V13. ^2^The P450 family gene seed file (PF00067) was downloaded from the Pfam online database. ^3^We used HMMER 3.0 (Eddy, 2011) software and the local BLASTP program to compare and search sequences containing the P450 protein domain. We uploaded the protein sequences to the online software Pfam, the Simple Modular Architecture Research Tool database (SMART),^4^ and the Conserved Domain Database (CDD).^5^ We performed sequence alignment and analysis to remove unannotated genes and redundant sequences. Finally, we obtained all members of the P450 gene family in four species and named the pepper P450 genes according to their positions on the chromosome. The amino acid number, molecular weight (MW), and isoelectric point (PI) were obtained from the ExPASy website,^6^ and the subcellular localization information was subsequently obtained from the CELLO website. ^7^ We downloaded the *Arabidopsis* P450 family gene members from the *Arabidopsis* genome database TAIR website. ^8^

### Phylogenetic and gene structure analysis

Because of the huge difference in the sequence of P450 proteins in pepper, we selected a protein length longer than 300 amino acids to construct the phylogenetic tree. We used Clustal X to perform multiple sequence alignments of the obtained genes from four species. The phylogenetic tree included multiple plant species and was constructed using MEGA 11 with the NJ method, and the selected model was the Poisson model with 1,000 bootstrap replications (Kumar et al., 2016). We used the ITOL^9^ web server to beautify the resulting phylogenetic tree. We used the software blastp to reclassify the proteins with a sequence length of less than 300 amino acids according to the constructed phylogenetic tree. We confirmed P450 protein motifs by the Multiple Expectation Maximization for Motif Elicitation (MEME)^10^ program with the motif number set to 10, and all the other parameters were set to default (Bailey et al., 2009). TBtools was used for visualization (Chen et al., 2020).

### Chromosomal localization, gene duplication, and synteny analysis

The chromosome physical location information of the pepper P450 gene family was extracted from the pepper genome annotation file information and visualized with TBtools. We analyzed the tandem duplication events of the P450 gene family using TBtools with MCScan X (Wang et al., 2012; Chen et al., 2020). In addition, we investigated segmental duplication events and the collinearity of gene pairs from different species using TBtools with the MCScanX and BLASTP methods (Wang et al., 2012; Chen et al., 2020). We calculated non-synonymous (Ka) and synonymous (Ks) substitutions between gene pairs using TBtools.

### Expression profile analysis of the P450 gene family in pepper

We downloaded the transcriptome data of pepper root, stem, flower, fruit development, and different periods under various viral infections (PRJNA223222), abiotic stresses (PRJNA525913), and hormone treatments (PRJNA634831) from the NCBI Sequence Read Archive (SRA) database (**Supplementary Table 1**) (Kim et al., 2014; Kang et al., 2020; Lee et al., 2020). We used the genome *C. annuum* cv. CM334 as the reference genome. We calculated the FPKM value (i.e., fragments per kilobase of the exon model per million mapped reads) using HISAT2 software for alignment and StringTie (v2.1.7) software for the assembly and quantitation with default parameters. We used log (FPKM+1) to calculate the expression level and imported the results into the R package “pheatmap” to draw the expression level of heatmap with the following parameters: scale = “raw,” cluster_rows = TRUE, and cluster_cols = FALSE.

### Quantitative analysis of candidate CaCYP genes

Total RNA was extracted from pepper leaves in various stages after cold and heat treatment of *C. annuum* cv. CM334 using TIANGEN’s polysaccharides- and polyphenolic-rich RNAprep pure plant plus kit. The online website Primer-Blast^11^ was used to design primers for the qRT-PCR experiment (**Supplementary Table 2**). We used HiScript IIQRT SuperMix from Vazyme to synthesize first-strand cDNA. We performed qRT-PCR with an ABI 7500 real-time PCR system using ChamQ Universal SYBR qPCR Master Mix from Vazyme. We also used the pepper ubiquitin-conjugating protein (CaUbi3) as a control for normalization between samples (Qin et al., 2018). We calculated relative transcript levels using the 2^−ΔΔCT^ method.

## Results

### Identification and phylogenetic analysis of the cytochrome P450 family genes in pepper

**Figure 1:**
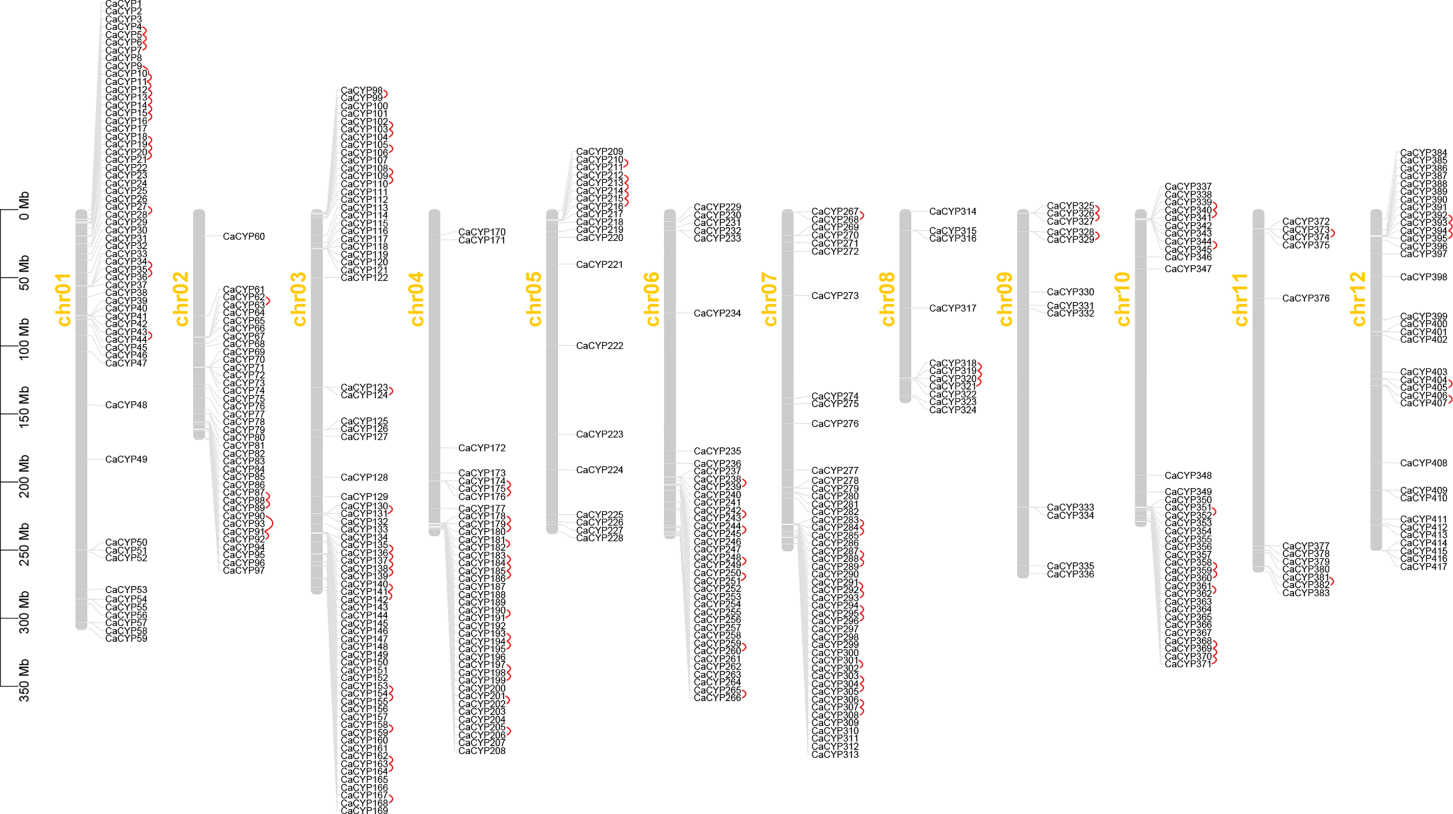
Chromosome distribution of the pepper P450 family, and 417 CaCYP genes are distributed on 12 pepper chromosomes. Chromosome number is indicated on the side of each chromosome. Tandemly duplicated genes are marked in red.

According to previous reports, the CYPome (i.e., the CYP complement of a given species) from angiosperms consists of approximately 300 genes (Nelson and Werck-Reichhart, 2011). In this study, we identified 478 P450 genes from the genome of *C. annuum* cv. CM334, which we named from *CaCYP1* to *CaCYP478* according to their location on chromosomes (**Figure 1**; **Supplementary Table 3**). In the identified proteins encoded by P450 genes, the smallest protein (CaCYP356) and the largest protein (CaCYP154) contained 53 and 1,321 amino acids, with molecular weights of 6.11 and 150.85 kDa, respectively. The theoretical isoelectric point of the coding protein ranged from 4.21 to 11.18. For subcellular localization, 11 CaCYP proteins were predicted to locate in the chloroplast, 203 CaCYP proteins in the cytoplasm, 10 CaCYP proteins in the extracellular membrane, 79 CaCYP proteins in the mitochondria, 32 CaCYP proteins in the nucleus, and 143 CaCYP proteins in the plasma membrane (**Supplementary Table 4**).

**Figure 2:**
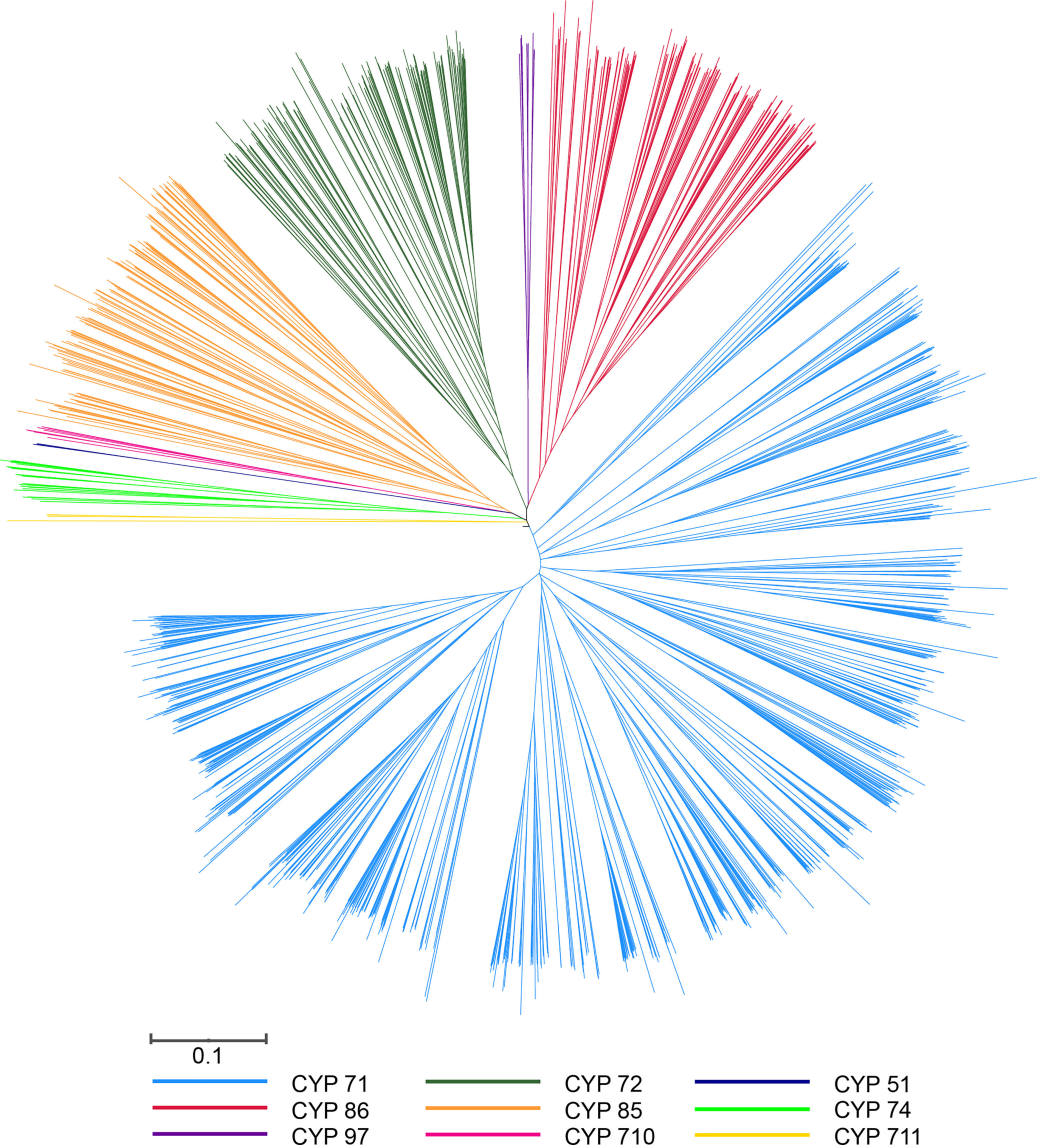
Phylogenetic tree of P450 genes from *Arabidopsis*, pepper, tomato, and potato. The unrooted tree was constructed by MEGA using the neighbor-joining (NJ) method with 1,000 bootstraps. The selected P450 proteins are clustered into nine clans and distinguished by different color lines.

Because of the significant difference in the length of the P450 genes in pepper, the phylogenetic tree could not successfully include all P450 genes. To investigate the evolutionary relationship of the P450 genes, 335 proteins were encoded by P450 genes for which a protein length longer than 300 amino acids was used to construct the phylogenetic tree. Finally, we constructed a phylogenetic tree containing 1,305 P450 genes from four species (*A. thaliana*, *C. annuum*, *S. lycopersicum*, and *S. tuberosum*) using the MEGA software (**Figure 2**; **Supplementary Figure 1**). We divided all of the P450 genes into one of the two major clades: A type, which contained the CYP71 clan; and non-A type, which contained eight clans. The remaining 143 CaCYP proteins with a length less than 300 aa were classified through comparison of protein sequence with the P450 protein used for the construction of the phylogenetic tree. Finally, we identified 478 CaCYP genes in the pepper genome. The number in each clan showed significant differences. For example, the CYP71 clan included 305 (63.81% of the total P450 genes in pepper) CaCYP genes, and the CYP51 or CYP711 clan contained only one (0.21%) of the CaCYP genes (**Supplementary Table 3**).

### Motif composition and gene structure of the CaCYP genes

To better understand their similarity and diversity, we investigated the conserved motif and gene structure using MEME software. We observed a total of 10 putative different motifs in the P450 genes of pepper (**Supplementary Figure 2**). Almost all of the P450 genes contained at least one motif and the greatest number of motifs was 17 (**Supplementary Figure 3**). We did not find, however, any motifs in the protein sequence of a few genes, including *CaCYP219*, *CaCYP354*, *CaCYP468*, and *CaCYP418*. Most of the members in the pepper P450 family contained eight motifs (motif 4, motif 7, motif 2, motif 6, motif 3, motif 5, motif 1, and motif 8) in sequence near the 3′ UTR regions. In addition, the motif composition of each subfamily member was different, but the motif composition of the same subfamily genes was similar. Moreover, compared with other subfamilies, we found that the CYP71 clan contained a specific motif 9. These results showed that the protein structure of each group of CaCYP genes was highly conserved. Except for motif analysis, we also analyzed gene structure and found that variations in the gene structure were also significant. The coding sequence (CDS) length of CaCYP genes ranged from 100 to more than 30,000 bp, and the number of introns or exons ranged from 1 exon and 0 introns to 16 exons and 15 introns, demonstrating that significant variation existed among the gene structure of CaCYP genes.

### Chromosomal localization and duplication events of the CaCYP genes

We identified 417 CaCYP genes distributed on 12 chromosomes, and another 61 CaCYP genes were unmapped scaffolds (**Figure 1**). Most of the CaCYP genes were concentrated at the terminal of each chromosome, with fewer exceptions of genes located in the intermediate of the chromosome. The number of CaCYP genes on each chromosome was slightly different, and the number ranged from 11 located on chromosome 8 to 72 located on chromosome 3.

**Figure 3:**
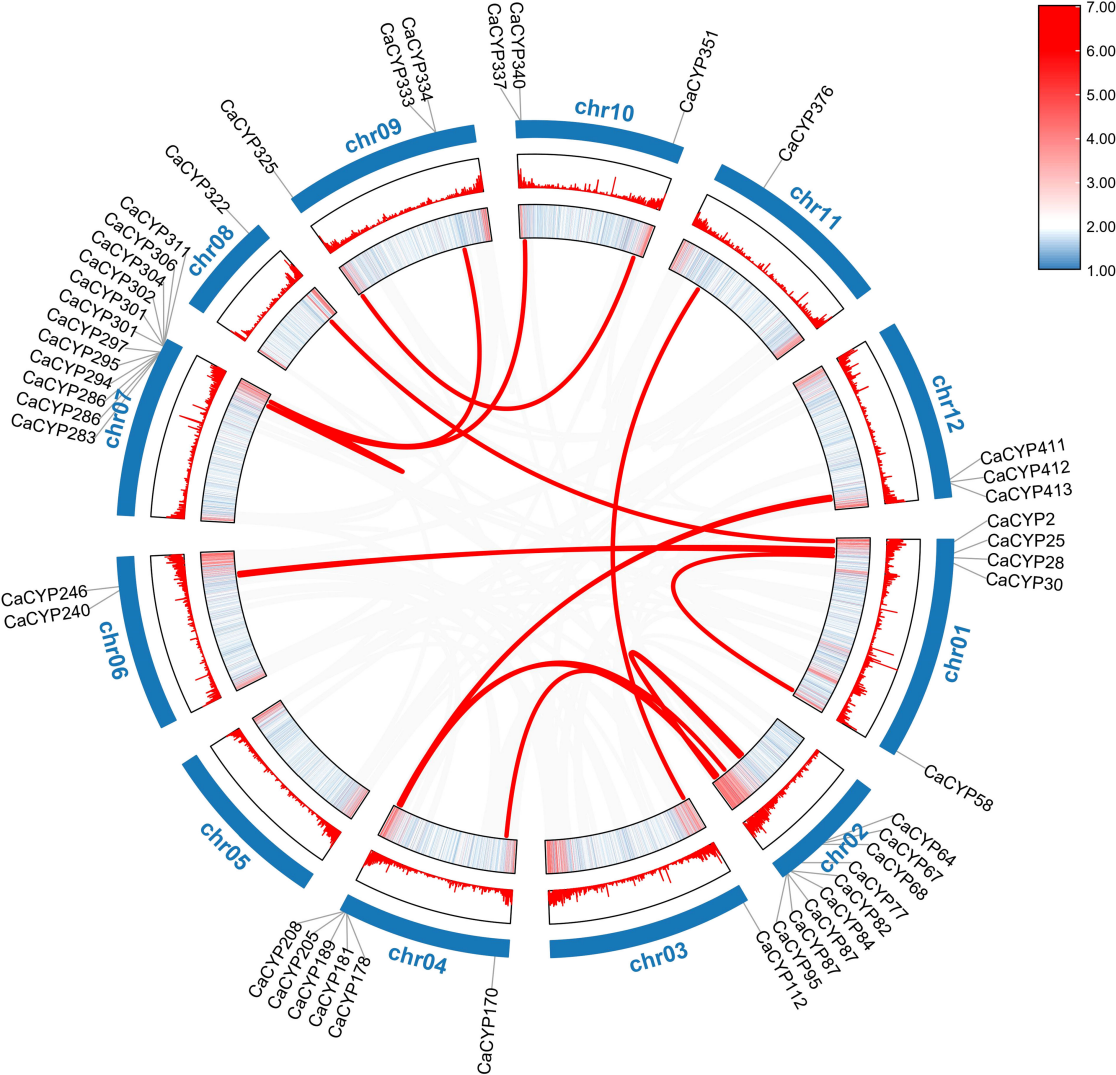
Collinearity analysis of the P450 family in pepper. The heatmap and lines represent gene density on the chromosomes. The CaCYP gene names of pepper were on the chromosomes. The gray lines in the background represent synteny blocks in the capsicum genome, while the red lines between chromosomes indicate segmental duplicated gene pairs.

According to the duplication event analysis at the genome-wide level, 106 pairs of tandem duplicated genes were discovered to be distributed across the 12 chromosomes (**Figure 1**). The number of tandem duplicated genes was different on each chromosome and 17, 5, 20, 15, 5, 7, 14, 3, 3, 10, 2, and 5 pairs of tandem duplicated genes were distributed on chromosomes from 1 to 12 (**Supplementary Table 3**). At the same time, we also observed 23 pairs of segmental duplicated genes on the pepper chromosomes (**Figure 3**; **Supplementary Table 5**). Moreover, we also evaluated the pressure of a selective constraint on each pair of duplicated CaCYP genes by calculating the Ka and Ks ratios (**Supplementary Table 6**). The results showed that every gene pair had a Ka/Ks ratio lower than 1, which indicated that the P450 gene family may have undergone strict purification selection during evolution. This result suggested that replicators were evolutionarily conserved and structurally stable and may have had consistent functions.

### Synteny analysis of CaCYP genes in pepper, tomato, potato, and *Arabidopsis*

**Figure 4:**
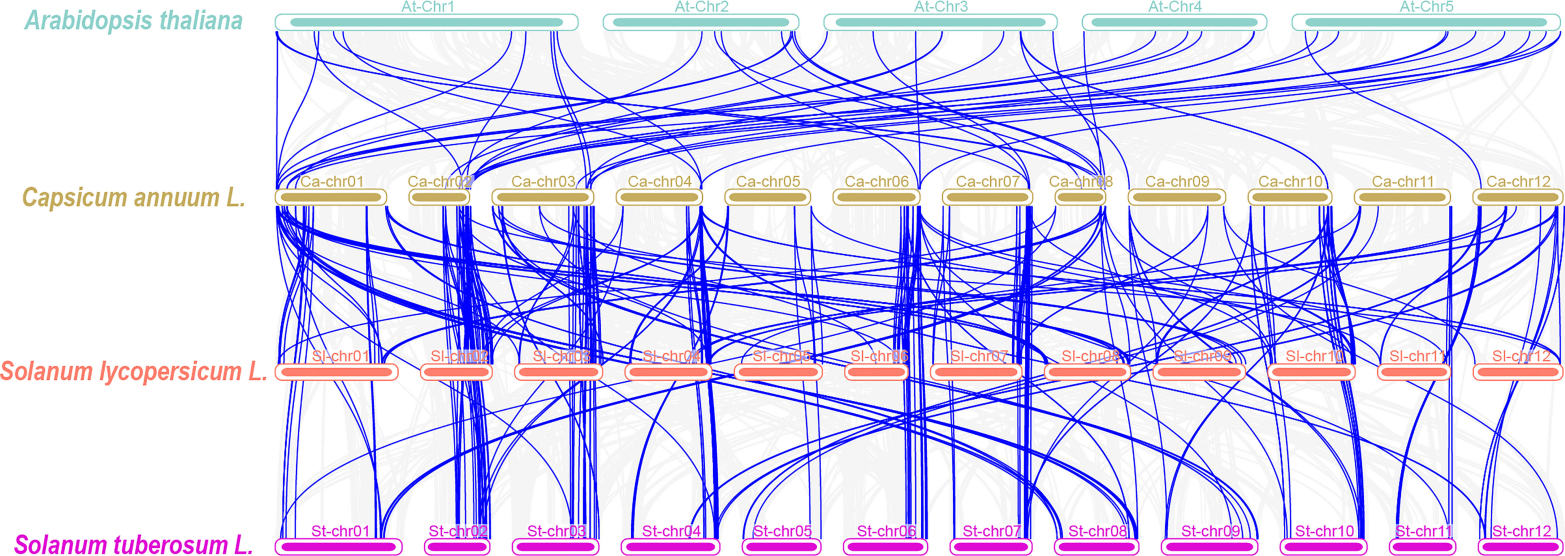
Synteny analyses between the CYP genes of pepper and three plant species. The gray lines in the background indicate collinear blocks within the capsicum genome and other three plant species genomes, while the blue lines highlight syntenic CYP gene pairs.

To further explore the evolutionary relationships of the P450 gene family, we analyzed the collinearity among pepper and other species (tomato, potato, and *Arabidopsis*) (**Figure 4**). According to the collinearity analysis between *Arabidopsis* and pepper, we identified a total of 50 pairs of genes, which indicated that the P450 gene family was significantly amplified before the differentiation of the two species. We observed 160 pairs of collinear genes between tomato and pepper and 178 pairs of collinear genes between potato and pepper (**Supplementary Table 7**). These results showed that the P450 genes of tomato or potato and pepper were highly evolutionarily conserved. Collinearity analysis showed that *CaCYP77* had four pairs of homologous genes and *CaCYP325* had three pairs of homologous genes between pepper and tomato. In pepper and potato, *CaCYP77* and *CaCYP325* each had three pairs of homologous genes. This result indicated that these homologous genes may have played a vital role in the evolutionary process. In addition, some pepper P450 genes matched one gene from potato or tomato, such as *CaCYP348*, *CaCYP325*, *CaCYP351*/*Soltu.DM.10G024690.1*, *CaCYP77*, *CaCYP178*, *CaCYP183*, and *CaCYP411*/*Solyc04g078270.3.1*, which indicated that these genes may have expanded during pepper evolution. Moreover, a large number of genes in three Solanaceae species (e.g., pepper, tomato, and potato) had the same homologous genes, indicating that these plants had great homology.

### Expression patterns of CaCYP genes in different tissues and in the fruit development stage in pepper

**Figure 5:**
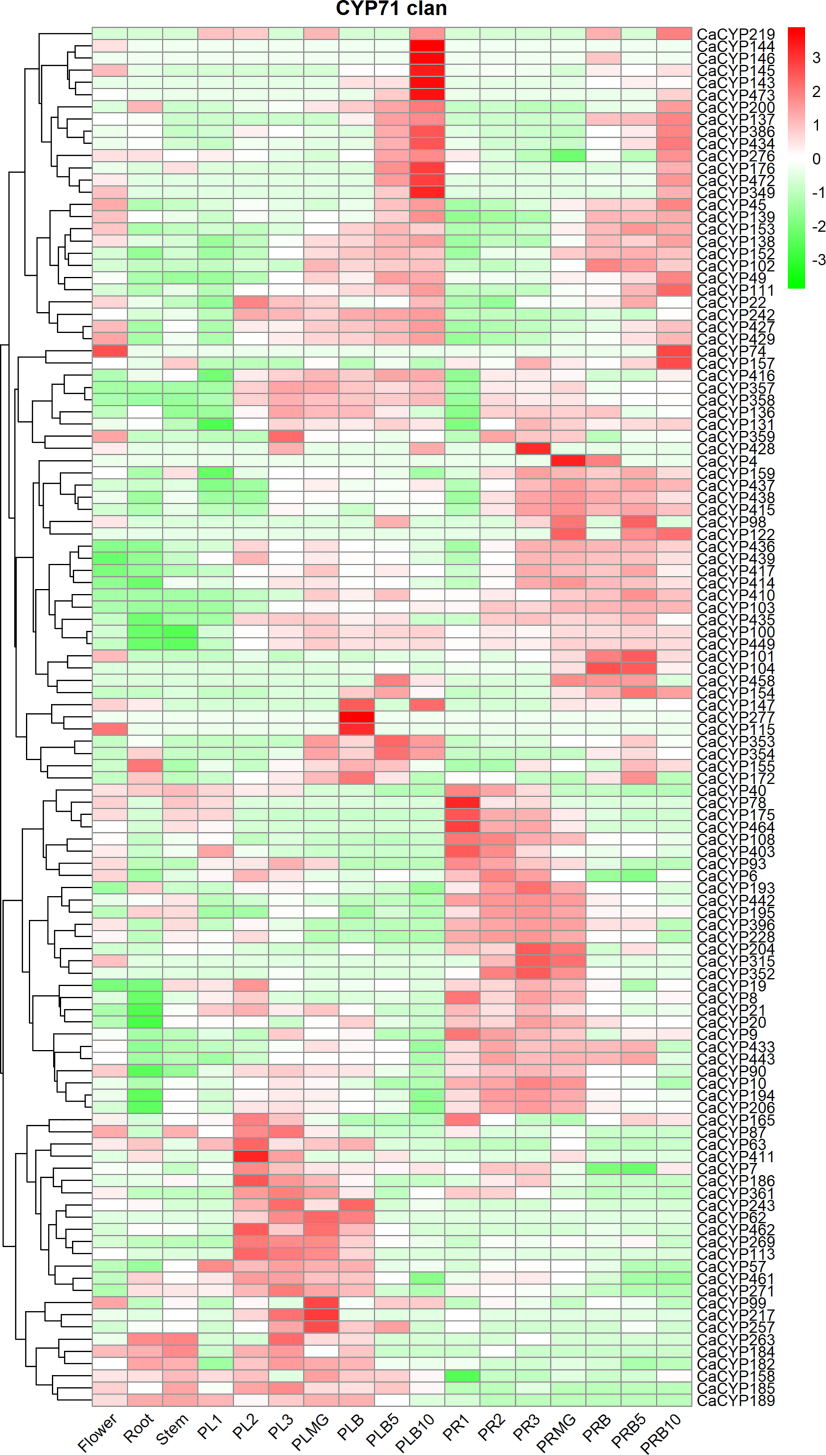
Analysis of the expression patterns of the selected CaCYP genes in different tissues and fruit development stages. Heatmaps of normalized RNA-seq data prepared from three biological replicates. The high-expression level is marked by red color, and the low-expression level is marked by green color. The different tissues containing flower, root, stem, placenta, and pericarp. PL1, PL2, PL3, PLMG, PLB, PLB5, and PLB10 represent placenta stage 1, placenta stage 2, placenta stage 3, placenta mature green, placenta breaker, placenta breaker plus 5 days, and placenta breaker plus 10 days, respectively. PR1, PR2, PR3, PRMG, PRB, PRB5, and PRB10 represent pericarp stage 1, pericarp stage 2, pericarp stage 3, pericarp mature green, pericarp breaker, pericarp breaker plus 5 days, and pericarp breaker plus 10 days, respectively.

We investigated the expression profile of CaCYP genes in various tissues and different fruit developmental stages according to published data (Kim et al., 2014). The results showed that in the CYP71 clan, some genes, such as *CaCYP57*, *CaCYP63*, *CaCYP186*, *CaCYP113*, and *CaCYP461*, showed high expression at placenta development before the fruit mature stage [at stage 1, stage 2, stage 3, and mature green (MG)], and some genes, such as *CaCYP40*, *CaCYP78*, *CaCYP175*, *CaCYP240*, *CaCYP315*, and *CaCYP352*, showed high expression at pericarp development before the fruit mature stage (at stage 1, stage 2, stage 3, and MG) (**Figure 5**; **Supplementary Figure 4**). In addition, we also observed high-expression genes both in the placenta and pericarp development after the fruit mature stage (breaker, breaker plus 5 days, and breaker plus 10 days) in the CYP71 clan, which showed that the CYP71 clan may have been significantly involved in fruit development. Except for the CYP71 clan, other clans also were found to be involved in this process. For example, *CaCYP56*, *CaCYP292*, *CaCYP295*, and *CaCYP294* in the CYP72 clan were related to placenta development after the fruit mature stage. We also found that some genes were related to pericarp development after the fruit mature stage. For example, *CaCYP310*, *CaCYP303*, *CaCYP305*, and *CaCYP309* in clan 72 were related to pericarp development before the fruit mature stage, whereas *CaCYP296*, *CaCYP273*, *CaCYP291*, and *CaCYP52* were related to pericarp development after the fruit mature stage. In the CYP74 clan, six CaCYP genes (66.7% of the total CYP74 clan) were related to the pericarp development before the fruit mature stage. In the CYP85 clan, *CaCYP456*, *CaCYP208*, *CaCYP173*, and *CaCYP222* were related to placenta development. *CaCYP95*, *CaCYP30*, *CaCYP275*, *CaCYP83*, *CaCYP51*, *CaCYP81*, and *CaCYP332* were related to pericarp development. Interestingly, all of the genes in the CYP97 clan were expressed at pericarp development, which showed that this clan may be connate for pericarp development.

### Analysis of the expression pattern of CaCYP genes under different abiotic stress

**Figure 6:**
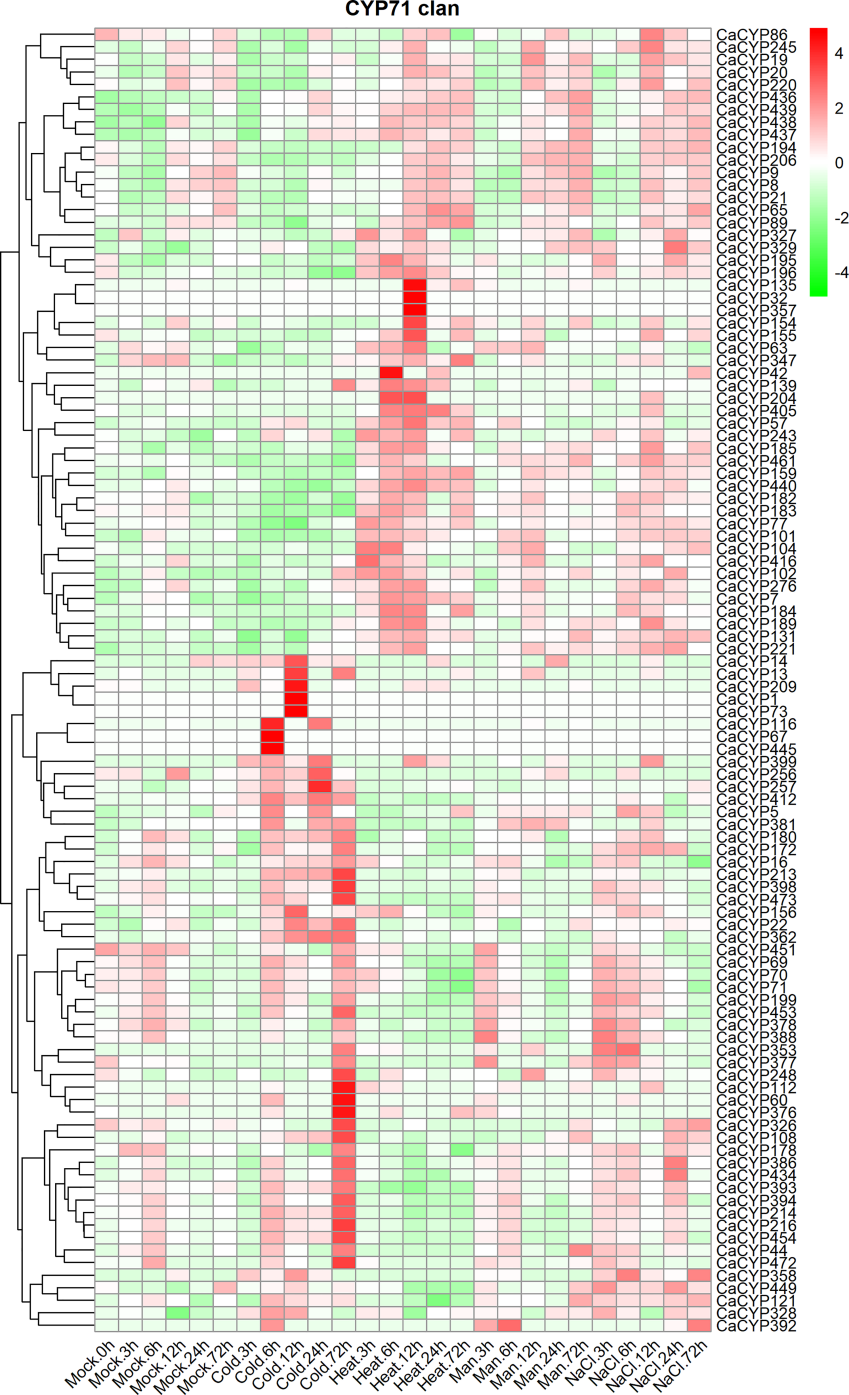
Analysis of the expression patterns of the selected CaCYP genes in different abiotic stresses. Heatmaps of normalized RNA-seq data prepared from three biological replicates. The high-expression level is marked by red color, and the low-expression level is marked by green color. Expression levels under abiotic treatment, including cold, heat, drought (Man), and salt (NaCl). Different time points include 3, 6, 12, 24, and 72 h. The control group is represented by the Mock tag.

**Figure 7:**
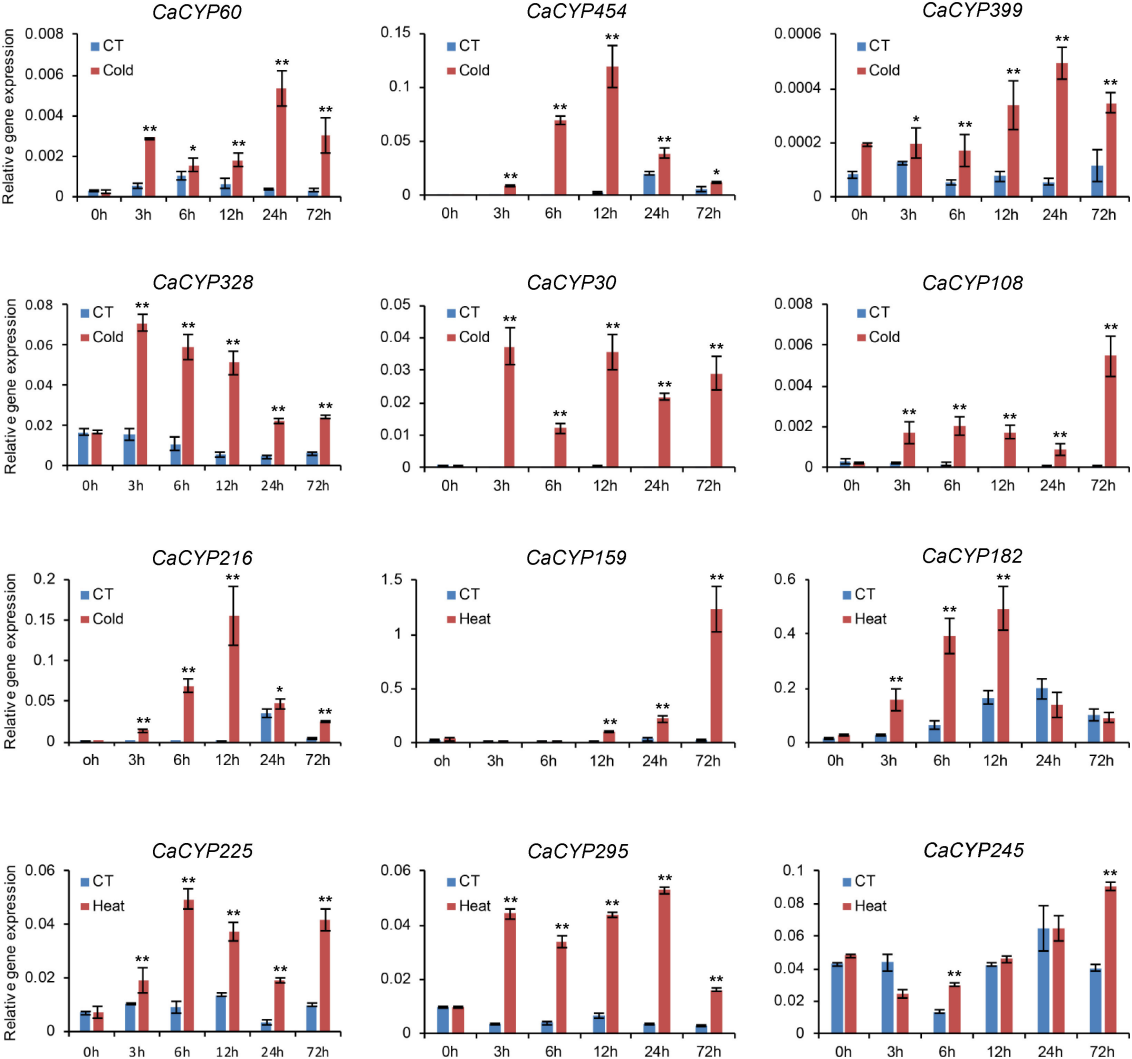
qRT-PCR analysis of the *CaCYP* gene expression under cold and heat stress. Leaves were picked up at 0, 3, 6, 12, 24, and 72 h after 10°C cold and 40°C heat treatments. Data represent the mean ± SD of three independent experiments. An asterisk on the bars indicates a significant difference by *t*-test. **P ≤ 0.01, *P ≤ 0.05 in *t*-test.

To further explore the function of CaCYP genes, we analyzed the RNA-seq data of pepper leaves after cold, heat, sodium chloride (NaCl), and D-mannitol treatment for 0, 3, 6, 12, 24, and 72 h (Kang et al., 2020) to explore the function of CaCYP genes in abiotic stresses. Most of the CaCYP genes were expressed under the four abiotic stresses, indicating that the CaCYP genes were closely related to abiotic stress (**Figure 6**; **Supplementary Figure 5**). In the CYP71 clan, 70 genes had high-expression levels under cold treatment, of which 11 genes had specific high expression at 72 h cold treatment, and 49 genes were expressed at almost all time points of cold treatment. Additionally, *CaCYP15*, *CaCYP137*, *CaCYP316*, *CaCYP219*, and *CaCYP325* had higher expression at 3 h of heat treatment. *CaCYP135*, *CaCYP32*, *CaCYP357*, and *CaCYP154* had higher expression at 12 h of heat treatment. *CaCYP114*, *CaCYP115*, *CaCYP106*, *CaCYP422*, *CaCYP62*, and *CaCYP120* were highly expressed at 12 h after D-mannitol treatment, whereas *CaCYP186*, *CaCYP130*, *CaCYP263*, *CaCYP462*, *CaCYP421*, and *CaCYP364* were relatively highly expressed under NaCl treatment. In addition, a total of 16 genes in the CYP72 clan were associated with cold treatment, and most of the remaining genes were expressed under the other three abiotic stresses. Three genes were related to cold treatment and five genes were associated with D-mannitol treatment and NaCl treatment in the CYP74 clan. In the CYP85 clan, eight genes were related to cold treatment, 13 genes were related to heat treatment, and seven genes had high expression levels in D-mannitol treatment and NaCl treatment. In the CYP86 clan, 10 genes were associated with cold stress, whereas *CaCYP465*, *CaCYP128*, and *CaCYP466* had very high expression levels under NaCl treatment. Finally, we found that some genes had similar expression patterns under heat, D-mannitol, and NaCl treatment, indicating these genes may have been involved in the production of cellular secondary metabolites to regulate cellular osmotic pressure. Moreover, we validated the RNA-seq results by qRT-PCR analysis of 12 CaCYP genes that responded to cold and heat treatment of pepper P450 genes. These selected genes were analyzed for relative transcript abundance (**Figure 7**). The results showed that the expression trend of these genes was highly consistent with that of RNA-seq analysis. All of these genes were highly expressed under cold or heat treatment. The expression levels of *CaCYP328* and *CaCYP30* were higher than those of the control after cold stress at 3 h. Heat stress induced the expression of *CaCYP225* at 6 h. *CaCYP454*, *CaCYP216*, and *CaCYP182* showed the highest expression levels after 12 h of cold or heat stress. *CaCYP60*, *CaCYP399*, and *CaCYP295* transcripts were elevated in response to cold and heat treatment at 24 h. Finally, *CaCYP108*, *CaCYP159*, and *CaCYP245* showed the highest levels after 72 h of cold or heat stress. These results indicated that the P450 family genes may play an important role in stress tolerance in pepper.

### Analysis of the expression pattern of CaCYP genes in different phytohormone treatments

**Figure 8:**
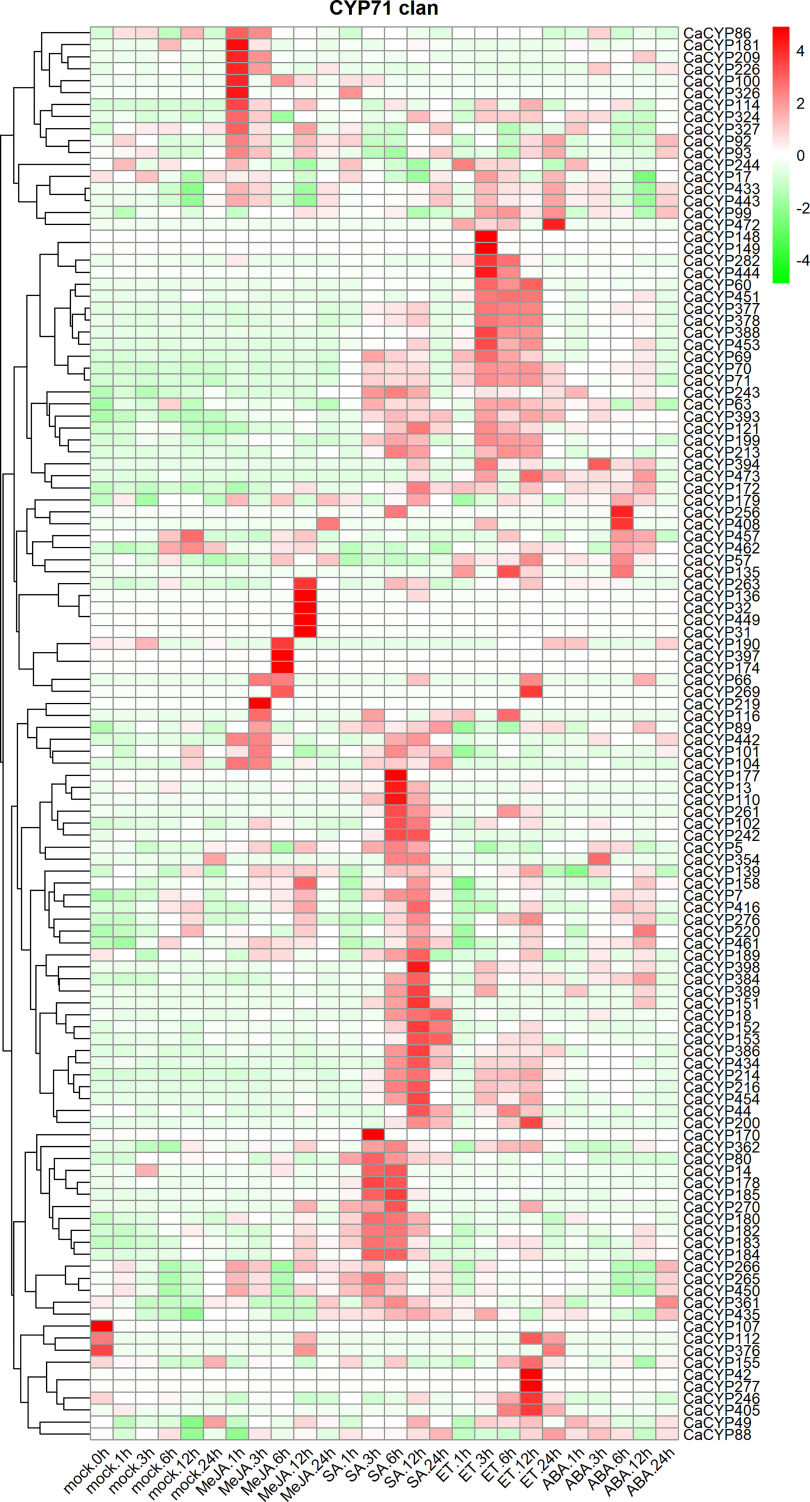
Analysis of the expression patterns of the selected CaCYP genes in different phytohormone treatments. Heatmaps of normalized RNA-seq data prepared from three biological replicates. The high-expression level is marked by red color, and the low-expression level is marked by green color. Expression levels after phytohormone treatment, including methyl jasmonate (MeJA), salicylic acid (SA), ethylene (ET), and abscisic acid (ABA). Different time points include 1, 3, 6, 12, and 24 h. The control group is represented by the Mock tag.

To further explore the function and expression pattern of CaCYP genes, we analyzed the RNA-seq data of pepper leaves treated with four hormones, including methyl jasmonate (MeJA), salicylic acid (SA), ethylene (ET), and abscisic acid (ABA) (Lee et al., 2020) (**Figure 8**; **Supplementary Figure 6**). High expression levels of CaCYP genes were observed under four hormone treatments. In the CYP71 clan, 11, 5, and 6 CaCYP genes had extremely high expression at 1, 6, and 12 h of MeJA treatment, respectively. We identified 52 genes related to SA treatment, of which 11 genes had higher expression levels during the early stage, 30 genes had higher expression levels during the late stage, and the remaining 11 genes had higher expression levels in every stage after SA treatment. A total of 56 genes were upregulated after ET treatment. Among them, *CaCYP244*, *CaCYP28*, and *CaCYP64* had higher expression levels at 1 h. *CaCYP22*, *CaCYP325*, *CaCYP137*, *CaCYP167*, *CaCYP101*, *CaCYP106*, and *CaCYP422* had higher expression levels at 6 h. *CaCYP155*, *CaCYP42*, *CaCYP277*, *CaCYP246*, and *CaCYP405* had higher expression levels at 12 h. A total of 17 genes were associated with SA in the CYP72 clan, and five and seven genes were associated with MeJA in the CYP74 clan and CYP86 clan, respectively. In addition, eight genes of the CYP85 clan were associated with ET. Moreover, 25 genes also were related to ABA treatment from 3 to 12 h, including *CaCYP378*, *CaCYP384*, *CaCYP453*, and *CaCYP69*. The analysis results showed that the P450 family genes responded to stress resistance-related hormones, which may be involved in the stress tolerance of pepper.

### Analysis of the expression patterns of CaCYP genes in virus infections

**Figure 9:**
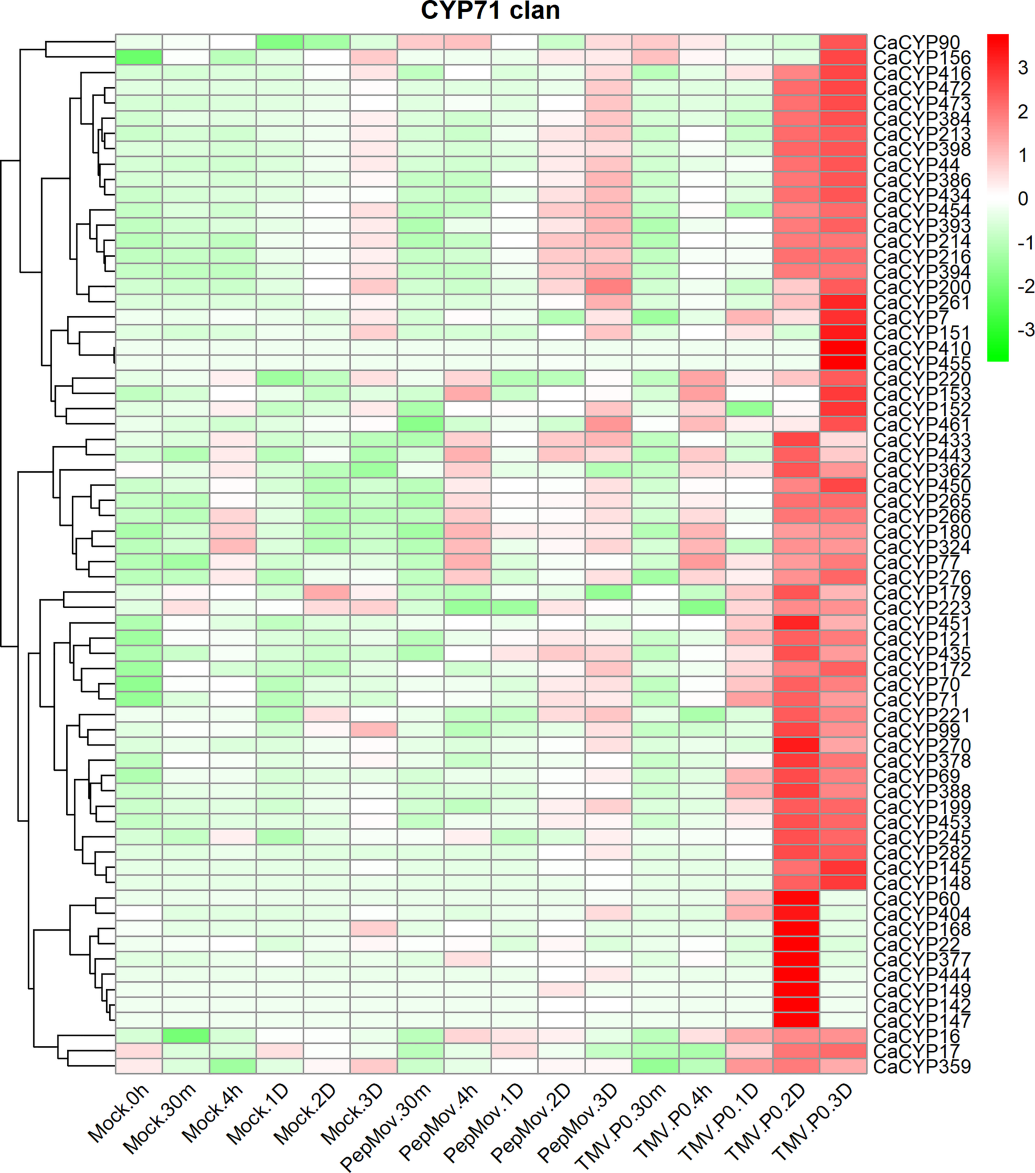
Analysis of the expression patterns of the selected CaCYP genes in different virus infections. Heatmaps of normalized RNA-seq data prepared from three biological replicates. The high-expression level is marked by red color, and the low-expression level is marked by green color. Expression levels after virus treatment, including tobacco mosaic virus (TMV) and pepper mottle virus (PepMoV). Different time points include 30 min, 4 h, 1 day, 2 days, 3 days, and up. The control group is represented by the Mock tag.

To explore the potential function of CaCYP genes under biotic stress, we analyzed the RNA-seq data of leaves infected with the tobacco mosaic virus (TMV) P0 strain and pepper mottle virus (PepMoV) at different infection times (Kim et al., 2014) (**Figure 9**; **Supplementary Figure 7**). The results showed that in the CYP71 clan, 68 genes showed slightly higher expression after the virus infected the leaves for 2 and 3 days compared with the control. Interestingly, these genes showed a strong response to TMV after the virus infected the leaves for 2 and 3 days. Except for the CYP71 clan, other clans also showed higher expression after TMV and PepMoV infection, including a moderate response to PepMoV after incubation; however, these genes showed a strong response to TMV. In the CYP72 clan, approximately 17 CaCYP genes significantly increased in the presence of virus infection, among which the expression level was higher in TMV infection. Eight CaCYP genes were expressed only in the presence of PepMoV infection. Three genes in the CYP85 clan showed higher expression levels after 2 and 3 days of infection. The results showed that the P450 family genes may be closely related to plant disease resistance.

## Discussion

Plant cytochrome P450 genes are involved in the catalysis of multiple reactions, including growth, development, and secondary metabolite biosynthesis pathways (Ikezawa et al., 2003; Glawischnig, 2006; Rai et al., 2015; Kinzler et al., 2016). In this study, we identified a total of 478 P450 genes in the pepper genome, of which 417 genes were mapped to chromosomes. Similar to the cluster analysis results of Tartary buckwheat and *Arabidopsis*, we divided the pepper P450 gene family into two major types of subfamilies: A type and non-A type. The two types of subfamilies were further divided into nine subfamilies (Sun et al., 2020). The A-type subfamily contained the CYP71 clan, which included most of the P450 genes. The first plant P450 gene was isolated from a ripening avocado and belonged to the CYP71A subfamily (Bozak et al., 1990). The CYP97, CYP710, CYP51, and CYP711 clans contained few genes compared with the other clans in pepper. The CYP97 clan had three genes and the CYP710 clan had two genes. In addition, both the CYP51 and CYP711 clans contained only one gene.

The reason for this phenomenon is that these four clans are ancient single families. The CYP51 clan is one of the oldest and the most conserved eukaryotic P450 subfamilies across evolution within fungi, animals, and plants (Bak et al., 2011). The CYP51 clan usually contains single or low copies in plant genomes and has long been considered the prototype of stable and highly conserved P450 subfamilies. The CYP710 clan is also conserved throughout all plants, including green algae (Nelson et al., 2008). The CYP97 clan is one of the oldest plant-specific P450 families, with three conserved subfamilies, with each one having only one gene in the most sequenced land plant genomes (Nelson and Werck-Reichhart, 2011). The CYP711 clan emerged early in plant evolution, usually in single or low copies. Family clustering on the phylogenetic tree suggested a deeper relationship. For example, the CYP86 clan was adjacent to the CYP97 clan, CYP86 clan enzymes were based on fatty acids and alkanes as substrates, and the CYP97 clan was related to carotenoid metabolism. The transformation of carotenoids to fatty acids and alkanes suggested that the CYP86 clan may have evolved from the ancestors of the CYP97 clan. Similarly, the CYP51 and CYP710 clans were paired enzymes in sterol biosynthesis, and the CYP710 clan played an important role after the CYP51 clan, suggesting a possible connection between the two families (Nelson and Werck-Reichhart, 2011).

In the process of species evolution, the duplication of genes is considered to be the initial force for the evolution of genomes and genetic systems. These doubled genes provide the most primitive materials for the formation of new genes, which eventually form new functions. Tandem duplication is considered to be the main reason for the expansion of gene families in the genome, with the characteristic that many members of a family are distributed in the same intergenic interval or bell-ringing intergenic interval. Fragment duplications are caused by the reorganization of chromatin. Among the P450 gene families, the CYP71 clan has been growing since the early trigger of diversification and has produced a series of gene duplications at an accelerated rate. In higher plants, the CYP71 family represented more than half of the genes. In the pepper P450 gene family, a total of 106 pairs of tandem duplicated genes and 23 pairs of segmental duplicated genes were identified. This burst of gene duplication likely contributed to adaptation to specific ecological niches and speciation. Duplicated gene pairs that exhibit diminished function or perform different functions may lose their function in evolution. This phenomenon has also been observed in pepper, where the expression patterns of the duplicated CaCYP gene pairs were different and had different functions. *CaCYP9*, *CaCYP10*, *CaCYP11*, *CaCYP12*, *CaCYP13*, and *CaCYP14* composed a group of tandem duplicated genes. *CaCYP11* and *CaCYP13* were root-specific genes. *CaCYP9* and *CaCYP10* were expressed in the pericarp. *CaCYP12* and *CaCYP14* were flower-specific genes. *CaCYP250* and *CaCYP251* were also a pair of tandem duplicated genes. *CaCY250* was a flower-specific gene, and *CaCYP251* was expressed in the roots and stems.

In plants, the expression of some P450 genes was specific in different tissues. For example, the expression level in the roots and flowers with more saponins was significantly higher than that in other tissues. The expression map showed that pepper CaCYP genes had strong tissue specificity in the roots and flowers, especially in the CYP71, CYP72, CYP85, and CYP86 clans. Carotenoids are a class of important natural pigments commonly found in yellow, orange−red, or red pigments of animals, higher plants, fungi, and algae (Ngamwonglumlert et al., 2020). The CYP97 clan includes key enzymes in carotenoid pigment biosynthesis. From the tissue-specific expression map, we found that the CYP97 clan was highly expressed in the development of the pepper pericarp, and the highest expression was found in the pericarp mature green stage and pericarp breaker stage, which confirmed that the CYP97 clan was involved in carotenoid biosynthesis in pepper. Members of the CYP74 clan were involved in the metabolism of oxygen and lipids, including JA, which played a role in cellular signaling and defense (Wasternack and Feussner, 2018). CYP74A is responsible for the formation of the precursor of JA and its derivatives, an essential signal compound in plants (Hansen et al., 2021). At the same time, transient transformation and expression of the CYP74 clan has been observed in the floral organs of *A. thaliana*, especially in mature pollen (Bak et al., 2011). In pepper, the CYP74 clan was also expressed in flowers, and the expression profile results showed that the pepper CYP74 clan was involved in the JA biosynthesis pathway. CYP86s catalyzed the oxidation of fatty acids to a variety of oxidation derivatives, and they produced a wide range of precursors to form waterproof biopolymers, such as cutin and suberin, which are essential for water transport or prevention of water loss. At the same time, it has been reported that CYP86s have had a positive regulatory effect on the plant immune system and a strong defense against pathogens (Wang et al., 2020a). CaCYP86 clan genes were obviously expressed in the roots and stems and were related to cold, drought, heat, and salt tolerance. The genes in the CaCYP86 clan were also observed to have a high-expression level in TMV and PepMoV infection, which proved that the CaCYP86 clan was indeed related to plant immunity of pepper.

## Conclusion

The cytochrome P450 gene family is widely involved in biochemical reactions of various primary and secondary metabolites. In this study, we identified 478 P450 genes in pepper from the pepper genome. We classified these genes into A type and non-A type, which were further divided into nine subfamilies. Genes in the same subfamily had a similar genetic structure and conservative motif distribution pattern. Chromosomal localization provided a clear distribution on the chromosomes of pepper cytochrome P450 genes. Duplicated events analysis showed that the duplicated genes provided the initial force for the evolution of pepper P450 genes. The inferred syntenic relationships of P450 genes among tomato, potato, *Arabidopsis*, and pepper indicated that homologous genes may have played a vital role in the evolutionary process. Expression analysis showed the diverse expression patterns of P450 genes in pepper. They could be expressed under various tissues and organs, hormone treatment, and various biotic and abiotic stresses at different periods, which indicated the diversity of their functional regulation. This result provided a theoretical basis for further study of the molecular evolutionary mechanisms and potential functions of the P450 gene family in pepper.

## Data availability statement

The datasets presented in this study can be found in online repositories. The names of the repository/repositories and accession number(s) can be found in the article/**Supplementary Material**.

## Author contributions

ZZ, ZL, FG, YaH, and LF designed the research. ZZ, YuH, ZD, YZ, and WT performed the research. , ZD, XW, JL, and LW analyzed the data. YuH, ZZ, ZL, and FG wrote the manuscript. All authors contributed to the article and approved the submitted version.

## Footnotes

1
http://peppergenome.snu.ac.kr

2
https://phytozome-next.jgi.doe.gov/

3
http://pfam.xfam.org/

4
http://smart.embl-heidelberg.de

5
http://www.NCBI.nlm.NIH.gov/Structure/cdd/wrpsb

6
https://web.expasy.org/compute_pi/

7
http://cello.life.nctu.edu.tw/

8
https://www.arabidopsis.org/

9
https://itol.embl.de/

10
https://meme-suite.org/meme/tools/meme

11
https://www.ncbi.nlm.nih.gov/tools/primer-blast/
