# Supplementary material for: Phylogenomic analysis of cytochrome P450 multigene family and its differential expression analysis in pepper (Capsicum annuum L.)
Source: Front Plant Sci. 2022 Dec 6;13:1078377. doi: 10.3389/fpls.2022.1078377 (PMC9763298; doi:10.3389/fpls.2022.1078377)
Supplement: Supplementary file 8 [file DataSheet_1.pdf]

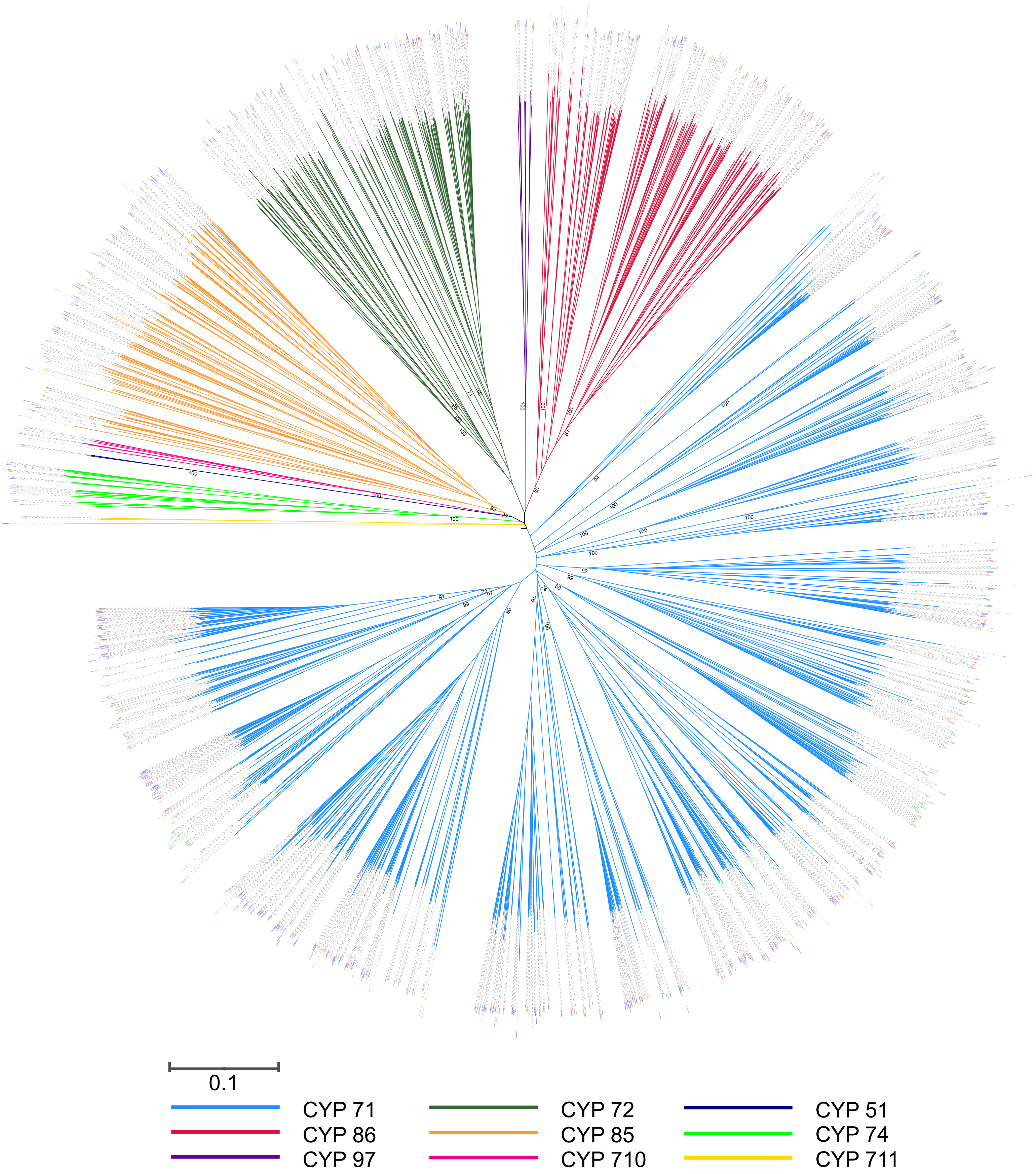

Supplementary Figure 1| Phylogenetic tree of P450 genes form 4 species. The gene in Arabidopsis are marked in green, the gene in capsicum are marked in red, the gene in tomato are marked in yellow, the gene in potato are marked in blue. The different color lines represents different clan.

Motif	E-value	Amino acid sequence composition of motif	Width
1	6.2e-2052	NDFEFJPFGAGRRICPGINLA	21
2	1.4e-1831	LPYLKAVIKETLRLHPPVPLL	21
3	2.7e-972	VNVWAIGRDPKIWEB	15
4	1.8e-1110	FAGTDTTSTTLEWAM	15
5	4.6e-879	PEEFKPERFLESDID	15
6	1.5e-859	EDCKVDGYTIPKGTR	15
7	2.8e-1112	ELLKNPEVMKKAQEEIRZVVG	21
8	9.0e-885	AVVELALASLLHHFDWKLPNG	21
9	8.5e-634	SYNGKDIAFAPYGPYWRQ	18

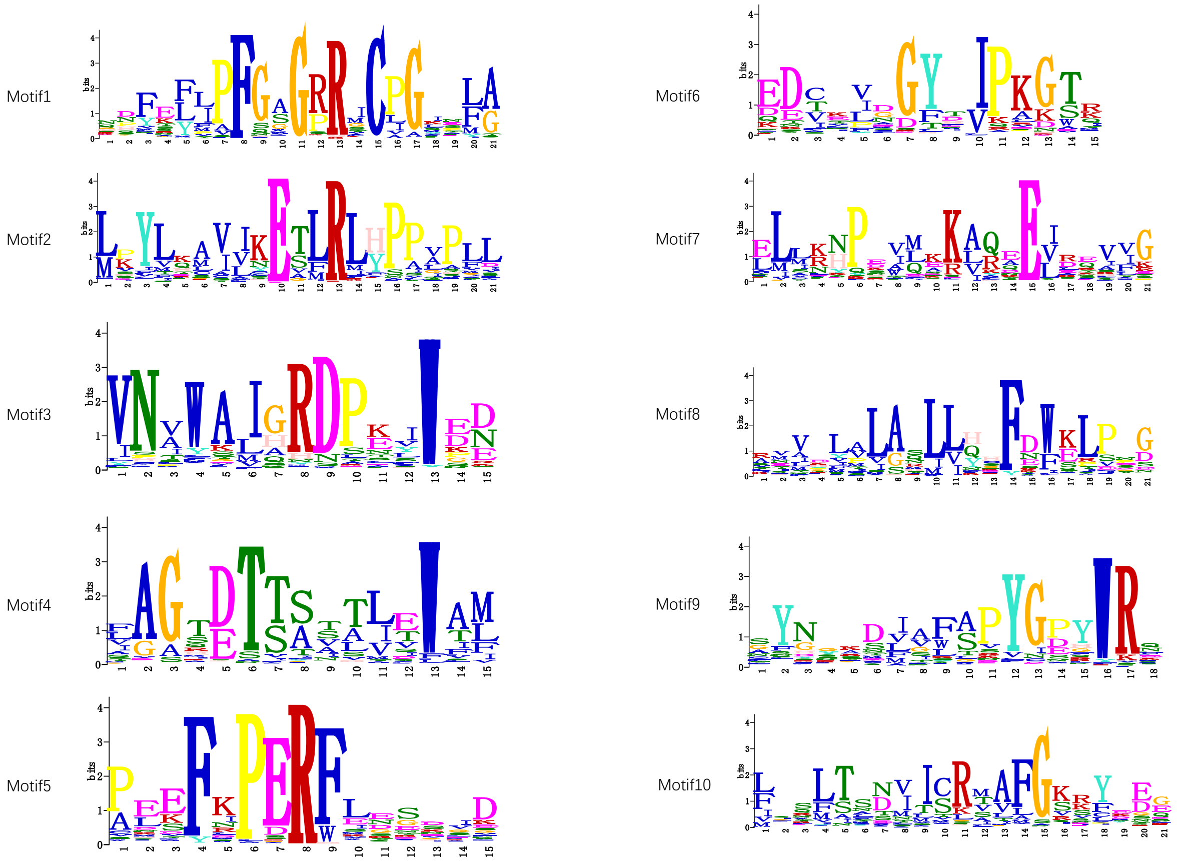

Supplementary Figure 2| The conserved motifs identified by MEME tools in CYP450 proteins.

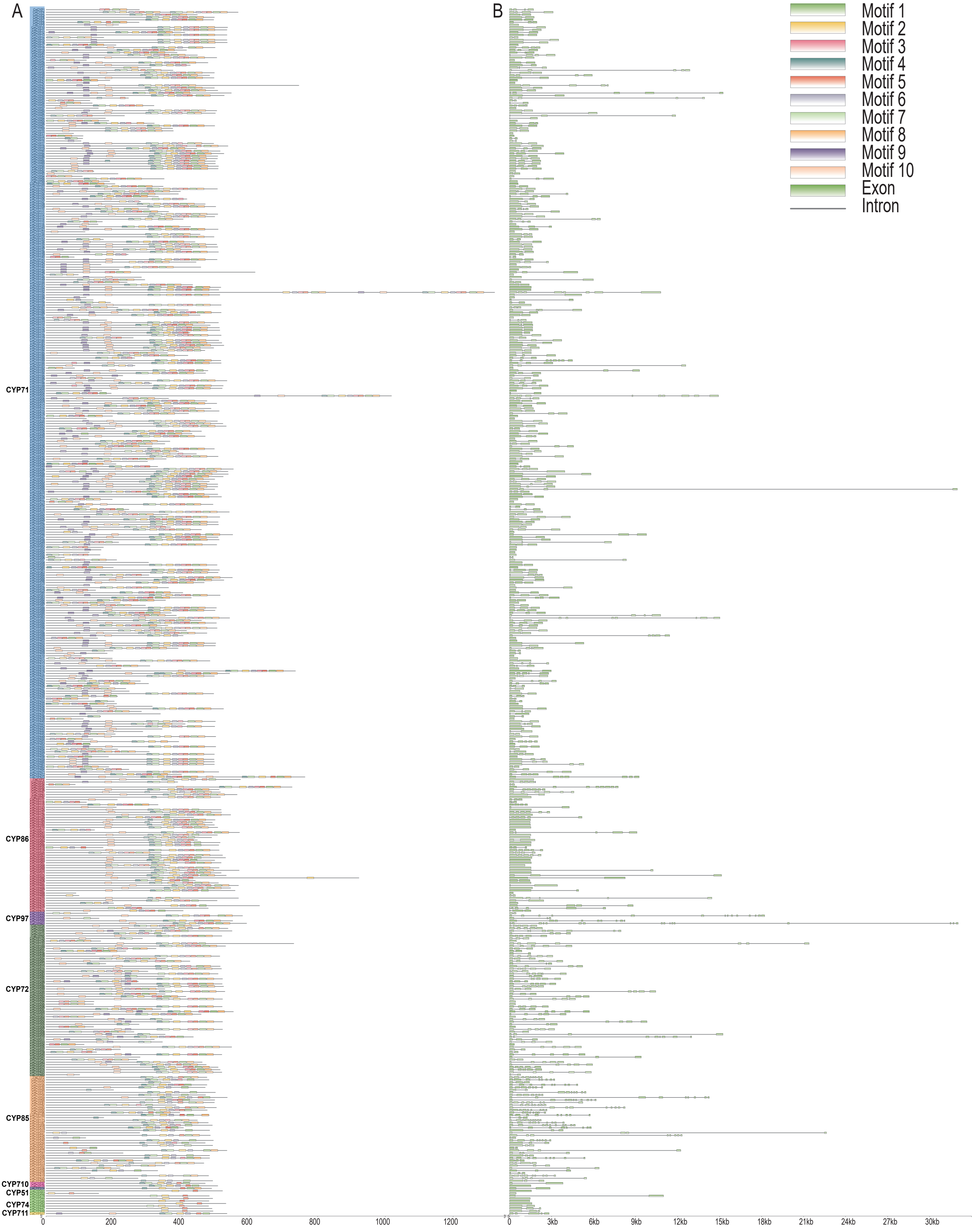
A
B
Motif 1
Motif 2
Motif 3
Motif 4
Motif 5
Motif 6
Motif 7
Motif 8
Motif 9
Motif 10
Exon
Intron
CYP71
CYP86
CYP97
CYP72
CYP85
CYP710
CYP51
CYP74
CYP711
0
200
400
600
800
1000
1200
0
3kb
6kb
9kb
12kb
15kb
18kb
21kb
24kb
27kb
30kb

Supplementary Figure 3| Conserved motifs and gene structure of the P450 family in pepper. (A) Subfamily classification and protein motifs of CaCYP members. The nine main subfamilies are marked with different colors. The colorful boxes represent different motifs. Motif sequence are provided in Supplementary Figure 2. (B) Analysis of Gene structure. Green blocks represent exon and black dashed lines represent intron.

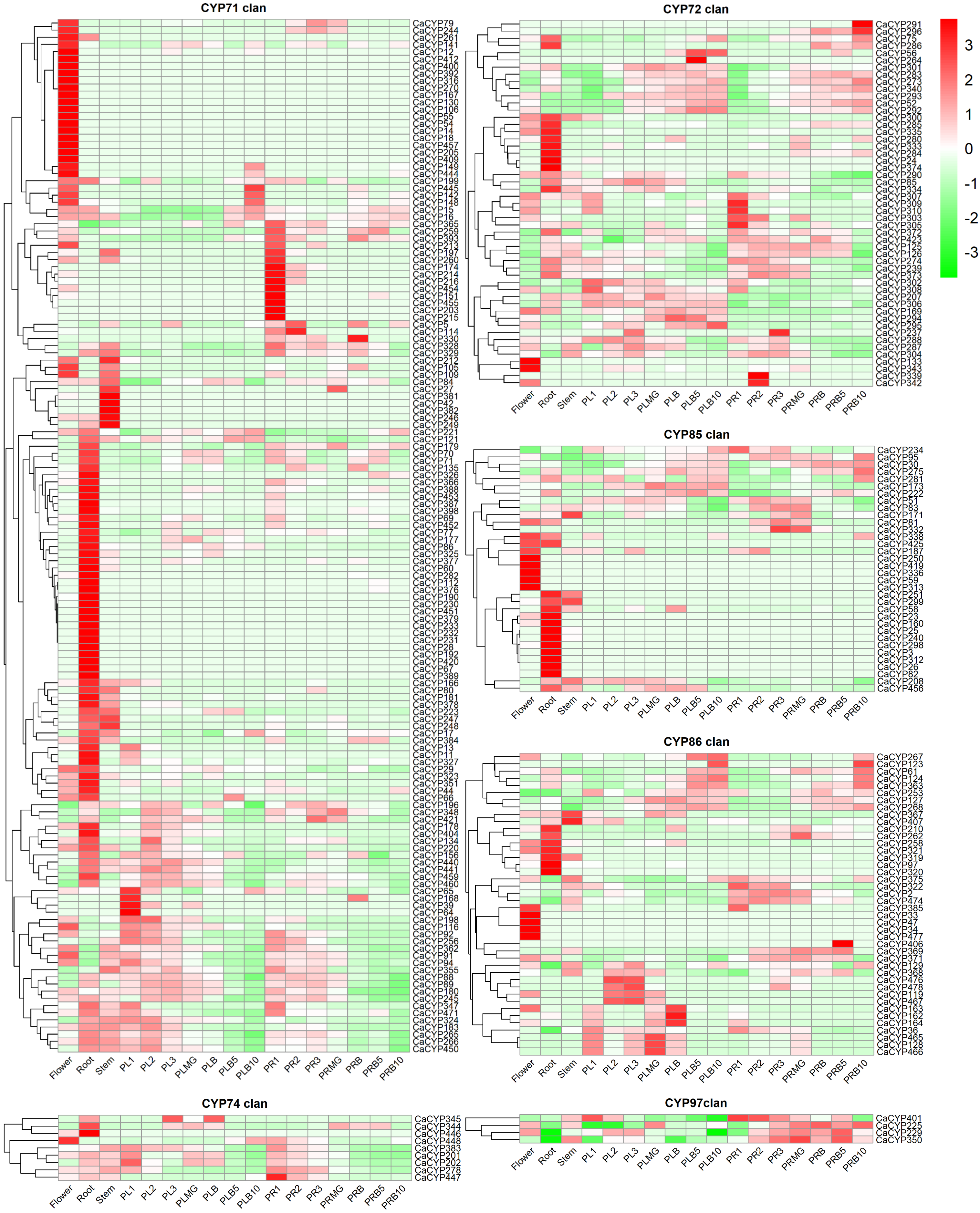

CYP71 clan
CYP72 clan
CYP85 clan
CYP86 clan
CYP74 clan
CYP97clan
Flower
Root
Stem
PL1
PL2
PL3
PLMG
PLB
PLB5
PLB10
PR1
PR2
PR3
PRMG
PRB
PRB5
PRB10
3
2
1
0
-1
-2
-3

Supplementary Figure 4| Analysis of the expression patterns of remaining CaCYP genes in different tissues and fruit development stages, which is corresponding to Figure 5.

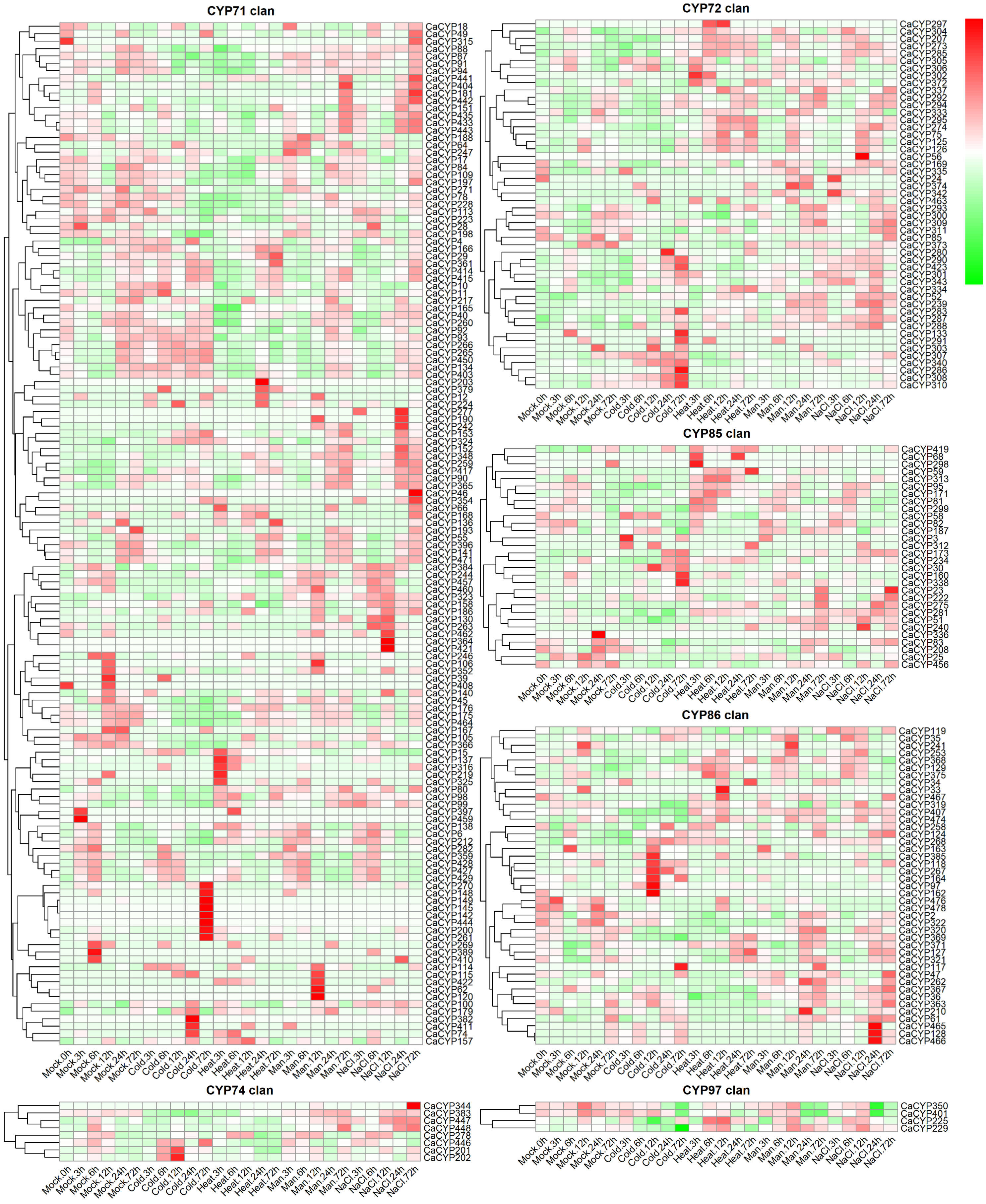
CYP71 clan
CYP72 clan
CYP85 clan
CYP86 clan
CYP74 clan
CYP97 clan
3
2
1
0
-1
-2
-3
Mock.0h
Mock.3h
Mock.6h
Mock.12h
Mock.24h
Mock.72h
Cold.3h
Cold.6h
Cold.12h
Cold.24h
Cold.72h
Heat.3h
Heat.6h
Heat.12h
Heat.24h
Heat.72h
Man.3h
Man.6h
Man.12h
Man.24h
Man.72h
NaCl.3h
NaCl.6h
NaCl.12h
NaCl.24h
NaCl.72h
CaCYP344
CaCYP383
CaCYP447
CaCYP448
CaCYP278
CaCYP446
CaCYP201
CaCYP202
CaCYP350
CaCYP401
CaCYP225
CaCYP229

Supplementary Figure 5| Analysis of the expression patterns of remaining CaCYP genes in different abiotic stress, which is corresponding to Figure 6.

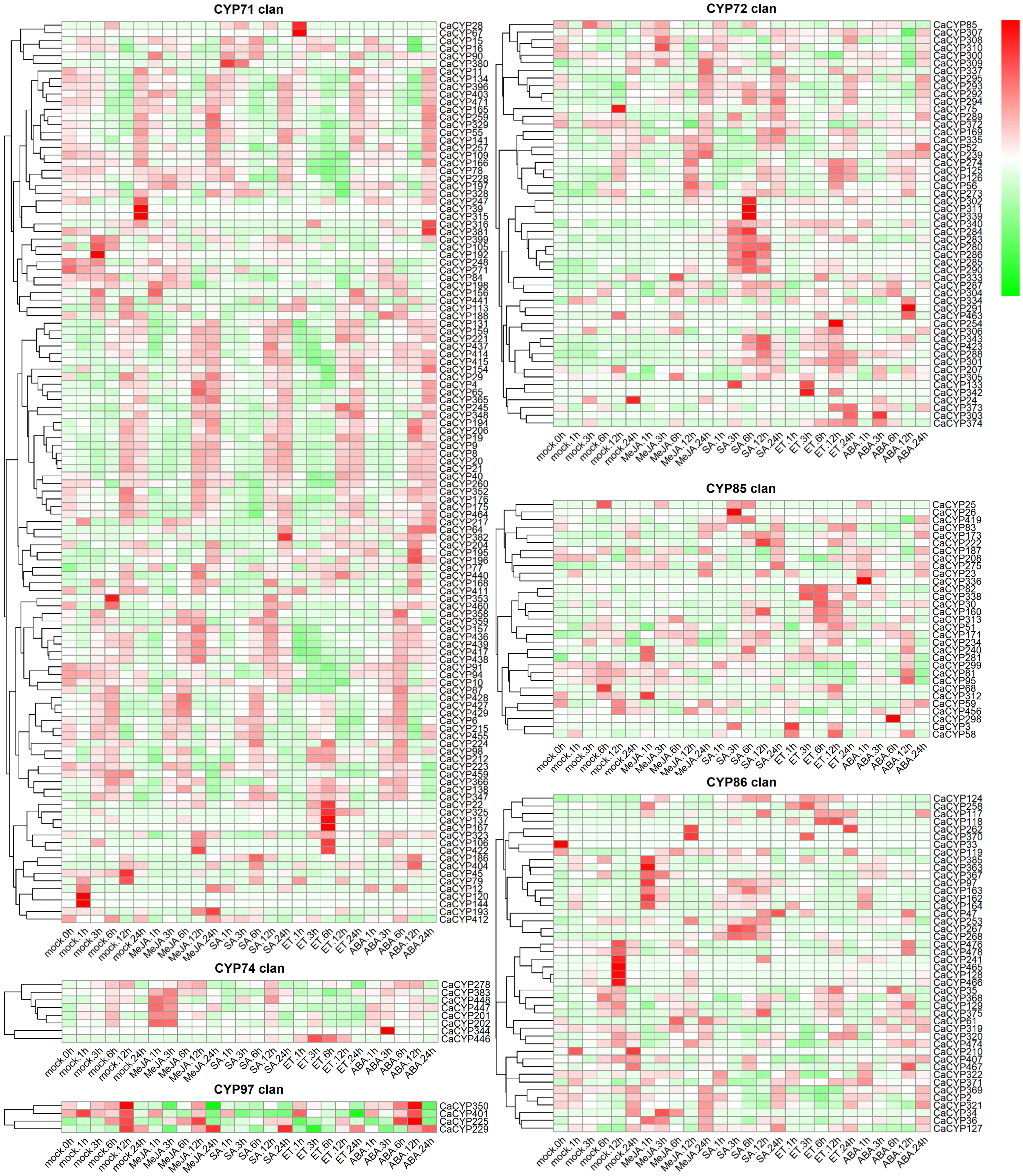
CYP71 clan
CYP72 clan
CYP85 clan
CYP86 clan
CYP74 clan
CYP97 clan
3
2
1
0
-1
-2
-3
mock.0h
mock.1h
mock.3h
mock.6h
mock.12h
mock.24h
MeJA.1h
MeJA.3h
MeJA.6h
MeJA.12h
MeJA.24h
SA.1h
SA.3h
SA.6h
SA.12h
SA.24h
ET.1h
ET.3h
ET.6h
ET.12h
ET.24h
ABA.1h
ABA.3h
ABA.6h
ABA.12h
ABA.24h

Supplementary Figure 6| Analysis of the expression patterns of remaining CaCYP genes in different phytohormone treatment, which is corresponding to Figure 8.

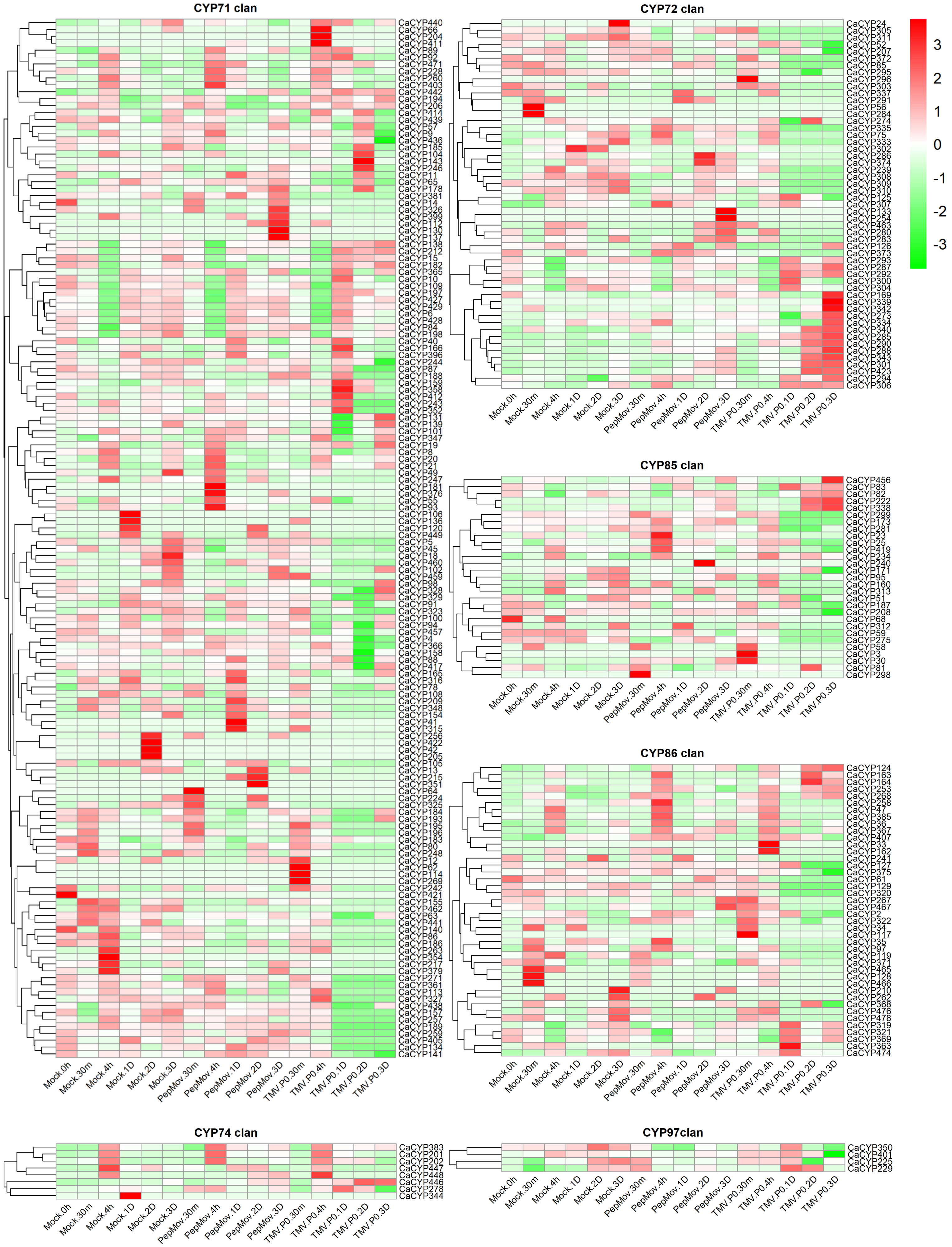
CYP71 clan
CYP72 clan
CYP85 clan
CYP86 clan
CYP74 clan
CYP97clan
3
2
1
0
-1
-2
-3
Mock.0h
Mock.30m
Mock.4h
Mock.1D
Mock.2D
Mock.3D
PepMov.30m
PepMov.4h
PepMov.1D
PepMov.2D
PepMov.3D
TMV.P0.30m
TMV.P0.4h
TMV.P0.1D
TMV.P0.2D
TMV.P0.3D

Supplementary Figure 7| Analysis of the expression patterns of remaining CaCYP Genes in different virus infections, which is corresponding to Figure 9.
